# Sustainable healthy eating behaviour of young adults: towards a novel methodological approach

**DOI:** 10.1186/s12889-016-3260-1

**Published:** 2016-07-15

**Authors:** Zuzanna Pieniak, Sylwia Żakowska-Biemans, Eliza Kostyra, Monique Raats

**Affiliations:** Consumer and Sensory Research Institute Ltd, Sienna 55/9, 00-820 Warsaw, Poland; Department of Organization and Consumption Economics, Warsaw University of Life Sciences (SGGW), 02-787 Warsaw, Poland; Department of Functional Food, Ecological Food and Commodities, Warsaw University of Life Sciences (SGGW), 02-787 Warsaw, Poland; Food, Consumer Behaviour and Health Research Centre, University of Surrey, GU-2 7XH Surrey, UK

## Abstract

**Background:**

Food, nutrition and health policy makers are poised with two pertinent issues more than any other: obesity and climate change. Consumer research has focused primarily on specific areas of sustainable food, such as organic food, local or traditional food, meat substitution and/or reduction. More holistic view of sustainable healthy eating behaviour has received less attention, albeit that more research is emerging in this area.

**Methods/design:**

This study protocol that aims to investigate young consumers’ attitudes and behaviour towards sustainable and healthy eating by applying a multidisciplinary approach, taking into account economical, marketing, public health and environmental related issues. In order to achieve this goal, consumers’ reactions on interactive tailored informational messages about sustainable from social, environmental and economical point of view, as well as healthy eating behaviour in a group of young adults will be investigated using randomized controlled trial.

To undertake the objective, the empirical research is divided into three studies: 1) Qualitative longitudinal research to explore openness to adopting sustainable healthy eating behaviour; 2) Qualitative research with the objective to develop a sustainable healthy eating behaviour index; and 3) Randomised controlled trial to describe consumers’ reactions on interactive tailored messages about sustainable healthy eating in young consumers.

**Discussion:**

To our knowledge, this is the first randomised controlled trial to test the young adults reactions to interactive tailor made messages on sustainable healthy eating using mobile smartphone app. Mobile applications designed to deliver intervention offer new possibilities to influence young adults behaviour in relation to diet and sustainability. Therefore, the study will provide valuable insights into drivers of change towards more environmentally sustainable and healthy eating behaviours.

**Trial registration:**

NCT02776410 registered May 16, 2016.

## Background

Currently, food, nutrition and health policy makers are poised with two pertinent issues more than any other: obesity and climate change [1, 2]. According to the World Health Organization [3] overweight-related problems occur more often than malnutrition. Convincing evidence exist linking obesity and poor diet with cardiovascular diseases, cancer and diabetes [4, 5]. In addition to the challenge for overweight-related problems, the policy makers also need to consider the impact of the diet/over-consumption on the environment. The environmental contribution of the food sector to total greenhouse gas emissions (GHGE) is estimated at 15 to 31 % [6, 7]. GHGEs of different food groups vary widely; nevertheless meat and dairy make the greatest part to GHGEs in the diet [8, 9].

At present, literature on consumer behaviour is trying to apply a multidisciplinary approach, taking into account economical, marketing, public health and environmental related issues. Consumer research has previously focused on specific areas of sustainable food, such as organic food [10, 11], local or traditional food [12–14], ethical food purchases [15, 16] meat substitution and meat reduction [17–20]. Additionally, consumer attitudes, perception and behaviour towards healthy eating have been widely explored [21, 22]. However, a more holistic view of sustainable healthy eating behaviour has received less attention, albeit that more research is emerging in this area. Owen and co-authors [23] explored consumers’ response to sustainable food in a qualitative approach; whereas Clonan and co-authors [24] investigated consumers’ attitudes towards food packaging, production methods in UK in a quantitative approach. To our knowledge, consumers’ perception, their attitudes, knowledge and information need towards a sustainable healthy diet has not yet been explored in Poland. This research proposal aims at investigating these issues. Researchers need to add to the evidence base by evaluating the impact of interventions that incorporate both health and environmental sustainability objectives [25]. There is a need to change consumers’ behaviour to adopt a diet with lower GHGEs [2] while also taking the nutritional issues into account. Using methods that have to date not often been utilized in food-related research this project aims through four linked studies to meet the three main objectives:

1. to explore consumer attitudes towards sustainable healthy eating (Studies 1,2 and 3);
2. to develop an index measuring a sustainable healthy eating behaviour (Study 2);
3. to investigate consumers reactions on interactive tailored intervention about sustainable healthy eating behaviour in a group of Polish young adults (Study 3).

Main research questions that will be addressed focus on: (1) What are consumer’s attitudes and behaviour towards sustainable healthy eating? (2) How can the self-reported sustainable healthy eating behaviour be measured? (3) What are factors determining consumers’ reactions to messages about sustainable healthy eating? (4) What are consumers’ reactions on interactive tailored communication about sustainable and healthy eating behaviour?

### Study status section

The study is currently ongoing. It started in June 2014 and it is foreseen until the end of December 2016. Initiation of the randomized trial is scheduled to occur in the second quarter of 2016. This trial is approved and was registered through the Bioethical Committee of the National Food and Nutrition Institute on December 29th, 2015.

#### Significance of the project

Scarborough and co-authors [26] modelled the impact of reduced meat scenarios on both GHGEs and health outcomes. Their findings show that encouraging reduced consumption of meat and dairy products and substitution with (seasonal) fruits, vegetables and cereals might reduce both environmental impacts and deaths from chronic diseases. It is evident from the existing literature that current consumption patterns in developed countries contribute negatively to both climate change and obesity prevalence. Therefore, these issues have to be tackled together to ensure consistent dietary advice for consumers while avoiding any unintended consequences by addressing them separately [1, 2]. This concept, which can be termed as ‘sustainable diet’ is not new [27]. It is a complex issue and there are still many gaps in our understanding of what a sustainable diet might comprise of [28]. Consumer’s role and the consumption side of the supply chain have been identified to be crucial in improving healthy choices and achieving sustainability goals, however it’s not simple: there are some trade-offs that consumers’ may counter [29]. FAO [30] defined a sustainable diet as: “*those diets with low environmental impacts which contribute to food and nutrition security and to healthy life for present and future generations. Sustainable diets are protective and respectful of biodiversity and ecosystems, culturally acceptable, accessible, economically fair and affordable; nutritionally adequate, safe and healthy; while optimizing natural and human resources*”. Given the complexity of this concept people might in turn make people confused about the food that they need to consume every day.

There is no agreed definition of sustainable healthy diet. Some countries, such as Germany [31] and Sweden [32] have developed food choice guidelines for their citizens that integrate health and sustainability. These guidelines include choosing seasonal, local, and wherever possible, organic fruits and vegetables and advocate consuming less meat and fish and considering packaging. In the UK a campaign known as “Livewell 2020” has been launched to educate people about sustainable healthy eating behaviour. Six principles for a “healthy planet” have been introduced: eat more plants, waste less food, eat less meat, eat less processed food, eat more certified food and eat a variety of foods. To the author’s knowledge there is no measurement scale for people’s self – reported health and sustainable eating behaviour. Validated measurement scales for healthy eating exist, e.g. interest in healthy eating [33], healthy eating index [34]. Measurement scales for ecological behaviour, such as pro-ecological behaviour [35], green eating [36] and sustainability of food practices [37] also exist. This study aims to develop an index that measures a consumer’s sustainable healthy eating behaviour.

### New research designs in consumer science

Consumer research mostly uses a cross-sectional design to measure perceptions, attitudes, and their association with behaviour [38]. However, using an experimental design, such as a randomised controlled trial (RCT) has many advantages since it eliminates some biases, for instance “selection bias” (pre-existing differences), “omitted variable bias” and partly “publication bias” (supporting results that are statistically significant) [39]. A systematic review study in developed countries suggests that the omitted variable bias is a major problem when non-experimental methods are used [40]. There is increased evidence that interventions to change (also dietary) behaviour are enhanced by applying theories of behaviour and behavioural change in their development, implementation and evaluation phase [41, 42]. However, many previous interventions have failed to employ a theoretical model of behavioural change [43].

### Theoretically underpinned interventions

**Fig. 1:**
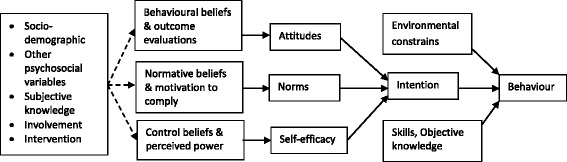
Theoretical model (adapted from integrative model [50]

The ecological, social and psychological sciences offer an understanding of why people engage in certain behaviours. Different social cognitive theories for explaining health behaviour exist, such as the Theory of Reasoned Action [44], the Theory of Planned Behaviour [45], the Health Belief Model [46, 47], the Protection Motivation Model [48], the Information-Motivation-Behavioural Skills model [49]. In this research proposal, a theoretical framework based on the Integrative Model [50] will be applied in order to identify the mediators and the factors that influence sustainable healthy eating behaviour. The Integrative Model combines several different leading theories of behavioural prediction and behavioural change [51–53]. It suggests that a behaviour is most likely to occur when one has a strong intention to act, has the necessary skills and abilities required to perform the behaviour and when there are no environmental constraints preventing behavioural performance. In turn, intentions are influenced by attitudes towards the behaviour (i.e. persons overall favourable or unfavourable feelings toward performing the behaviour), perceived norms concerning the behaviour (including both perceptions of what other people think one should do as well as perceptions of what others are doing), and self-efficacy with respect to performing the behaviour (i.e. person’s appraisal of their ability to perform a behaviour). The three determinants are themselves functions of underlying beliefs (see Fig. 1). Finally, the traditional demographic, personality, attitudinal and other individual variables may indirectly influence behaviour. In this research proposal, the model will be adapted by adding/highlighting a clear link with knowledge. There is little doubt that knowledge is an important factor in consumer behaviour [54, 55]. Previous studies have shown that knowledge is an important determinant of organic food consumption [10], fish consumption [56] and ecological behaviour [57, 58].

### Adaptive e-learning interventions

A recent systematic review of adaptive e-learning interventions for dietary behaviour change Harris and co-authors [59] reported that only one-third of the interventions that state to be theory-based measure theoretically predicted mediators; without this, the statement that the intervention is theory-based has limited scientific value. There are three main benefits of applying theory/theoretical framework. First, pertain to the identification of constructs that are hypothesized to be causally related to behaviour and are therefore appropriate targets for the intervention. Changing constructs that cause behaviour might lead to behavioural change [60]. Second, collecting empirical data within a theoretical framework facilitates the accumulation of evidence of effectiveness across different contexts, populations, and behaviours [61]. Third, theory-based interventions can evaluate the role of mediators and moderators that influence behaviour; thus may help to improve the understanding of why interventions are effective or ineffective [62, 63].

### Use of new technologies

New media such as the Internet and mobile telephones are recommended to provide means to achieve mass tailoring in topics related to healthy eating, physical activity and weight-related recommendations [64]. The existence of nearly 2.7 billion active mobile phones worldwide illustrates the huge potential for the mobile learning (mLearning) market [65]. Even though it is at an early stage, it is already drawing a great deal of attention in Asia, US and Europe [66]. mLearning enables the delivery of instructional content messages. The instructional messages can be designed in a way that they modify the cognitive, affective or psychomotor behaviour of a person through manipulation and planning of different symbols and signs [67].

Computer tailoring provides the opportunity to reach far more people at a lower cost [68] and at home [69] as compared with interpersonal countering [70]. According to the elaboration likelihood model of persuasion [71], personalised messages are more likely to motivate people to elaborate on the messages and to process information more deeply, and as a consequence to form attitudes that will be better predictors of consequent behaviour. In this proposal, we will use both experimental and structural methods to empirically test consumers’ reactions to tailored messages provided through mobile learning on consumers’ attitudes, self-efficacy and knowledge and at the end on sustainable healthy eating behaviour.

## Methods/Study design

**Fig. 2:**
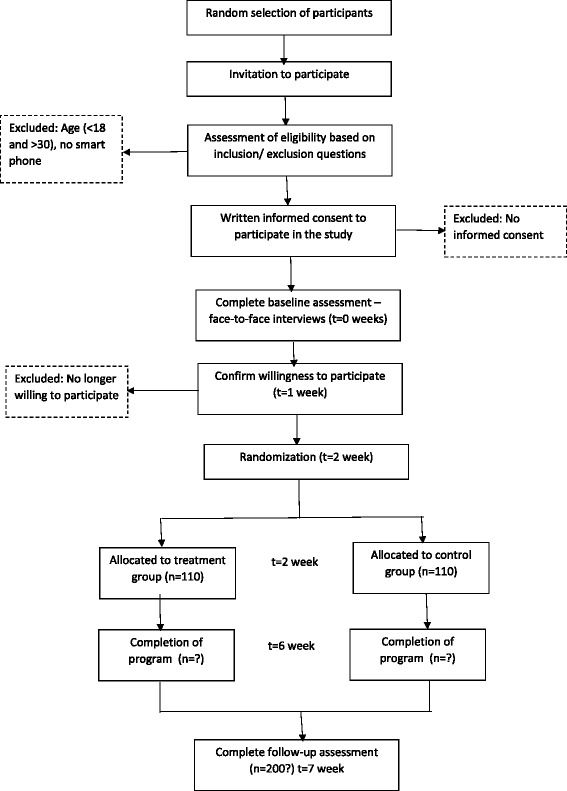
Study 3 flow diagram

To undertake the overall objective of the proposed research, the empirical research is divided into 3 studies:

1. Qualitative longitudinal research
  Qualitative longitudinal research (QLR) is a relatively recent development which has yet to be fully articulated as a coherent methodology [72]. It embodies a range of in-depth interviews which involve returning to interviewees to measure and explore changes which occur over time and the processes associated with these changes [73]. This novel approach is very relevant for this research proposal as sustainable healthy eating behaviour is not explored yet in Poland.
  Twenty interviews will be carried out in three time periods in order to get insights on consumers’ attitudes, knowledge and perception as well as current behaviour towards sustainable healthy eating. The selection of issues in the topic guide will be based on the outcome of the literature review. However, based on current understanding, the six principles introduced in the campaign “Livewell 2020” will be used as a starting point to discuss different elements of sustainable healthy eating with the interviewees. Participants will be recruited from the local area by advertisements made in local newspapers and on local websites. The target group of our intervention will be young adults (aged 18-30). Young adults often establish unfavourable dietary habits when leaving the parental home, i.e. consuming a diet of limited variety, high snacking, consuming more high-fat foods (including fast foods), more soft drinks, and less fruit and vegetables e.g. [74]. Such habits may have a long-lasting impact on their own health or the health of their future families [75, 76]. Young adults are also the future or current young parents. Therefore, it is important to explore the sustainable healthy eating behaviour to this particular group. The sample will consist of two groups: one more interested in sustainability issues and second consisting of people not specifically focused on sustainability issues. The three waves of interviews will be transcribed verbatim for subsequent analyses. Transcripts will be analysed systematically to capture three critical elements: time, process and change. Reporting of the findings from this qualitative study will adhere to COREQ guidelines [77].
2. Quantitative survey (SHE index)
  Although obesity and climate change are two of the hottest topics in the food-related research, up to date, there is no agreed definition of sustainable healthy diet. Validated measurement scales for healthy eating (e.g. interest in healthy eating [33], healthy eating index [34] and for sustainable behaviour (e.g. index of sustainability of food practices [37] exist. This study aims to develop an index that measures a consumer’s sustainable healthy eating behaviour.
  Based on the literature review and information collected through in-depth interviews a questionnaire will be developed, pre-tested and validated on a judgment sample of 200 young respondents in Warsaw and Mazovian region [78]. Judgment sample is a type of non-random sample, which is selected based on the opinion of the experts [79]. The preliminary questionnaire will be designed to assess a wide range of sustainable healthy eating behaviours such as: purchase of local foods, meat, dairy and plant consumption, portion size, processed food consumption, food packaging, seasonal food. SHE Index will be validated by means of convergent and discriminant validity as indicated by Hair and co-authors [80]. The developed measure will be used as an outcome measure in Study 3 as described below.
3. Randomised controlled trial
  Consumer research mostly uses a cross-sectional design to measure perceptions, attitudes, and their association with behaviour [38]. However, using an experimental design, such as a randomised controlled trial (RCT) has many advantages since it eliminates some biases, for instance “selection bias” (pre-existing differences), “omitted variable bias” and partly “publication bias” (supporting results that are statistically significant) [39]. A systematic review study in developed countries suggests that the omitted variable bias is a major problem when non-experimental methods are used [40].
  This research design assigns subjects randomly to either a study or a control group (Fig. 2). The study group experiences an intervention or experiment while the control group does not. Both groups are observed at two points in time, before (baseline) and after (follow-up) an intervention or experiment. The integrative model will be adapted after Study 1 (in-depth interviews to the specific case of sustainable healthy eating behaviour in Poland). As in Study 1 the target group of our intervention will be young adults (aged 18-30).

The content of the intervention build on the results of Studies 1 and 2 and will be assessed in a small pre-pilot. Around 10 participants from a convenience sample will be recruited for the pre-pilot. Feedback on the interventions will be collected by a short questionnaire with open-ended questions. Then, about 200 Polish consumers will be recruited for the pilot RCT. A stratified design will be used to recruit 18-30 years old adults using Internet and mobile phones for the intervention. The sample will be represented for gender and region. Recruitment of the sample as well as baseline and follow-up surveys will be done by a professional market agency in accordance with ICC/ESOMAR – International Code of Conduct for Market and Social Research. Highly qualified and experienced interviewers will carry out face-to-face interviews at home using a CAPI approach (Computer Assisted Personal Interviewing). Use of a professional market agency for recruitment and data collection is beneficial for the project in a way that the sample will be geographically wide, will include spread of socio-economic characteristics and data will be collected relatively fast. The sample will be randomly allocated into a treatment group and a comparison control group that never received the treatment as recommended by Schulz and Grimes [39]. Eligible participants will be randomised in a 1:1 ratio to either the intervention or control group, stratified by gender. A computer generated randomisation sequence with variable block sizes of 2 or 4 will be used. The group allocation will be concealed until the point of participant randomisation. Researchers will be blind to group allocation.

After the randomisation the participants will complete a baseline survey that includes the measure developed in Study 2 and extended by questions measuring different psychometric variables, such as consumer attitudes, norms, subjective and objective knowledge, involvement or self-efficacy (Fig. 1). The SHE index developed in Study 2 will be used as a dependent variable. A relative change in the SHE index will be used as a primary outcome. The intervention will be considered as successful (effective) when a meaningful change in the primary outcome will take place, i.e. the SHE index will increase by 5 %. We are aiming for a total sample size of 220 which will allow us to detect an effect size (measured by Cohen’s d statistic) of 0.44 for the SHE index. This would be considered a ‘medium’ effect size. The data collected in the pilot trial will allow us to refine our sample size estimates for the main trial.

A segmentation analysis will be carried out on consumers’ knowledge, attitudes and dietary habits from the baseline survey in order to obtain different groups of consumers who will receive tailored messages.

Tailoring messages has been proven to be an effective health education approach [81, 82]. Research in computer-mediated health education has indicated that adapting and tailoring health content to the individual can increase their engagement, improve leaning, foster positive attitudes about behaviour change and enhance health intentions e.g. [82–84]. Additionally, there is evidence that computer-tailored nutrition education is a more effective tool for motivating people to change to healthier diets than general nutrition education [70].

An application for smart phones will be developed by a professional IT company in order to deliver personalised messages to the participants. The design of such a delivery tool for mobiles is highly innovative. Furthermore, the intervention includes an element of “interaction” with the consumers via the mobile which is highly original. Messages will be created by the researchers before the start of the intervention. Specialised software to send the intervention at the correct time will be used. Once the intervention is sent, the links will be automatically modified to track the user who clicks on them. Software will be written which sits on the content pages, records when a particular user visits the page, along with the time that the user spends on the page. The participants will be able to go back to the previous messages. Messages will be sent daily or every 2–3 day depending on the results from the pre-pilot study in order to avoid annoying consumers. When receiving a message a push notification will be used. Push notifications in an app is the most effective means of interrupting participants and persuading them to read a message.

Messages will be developed following the theory-linked definitions of behaviour change techniques (BCT) [85]. In the intervention different BCT will be used which would support the theory presented in the integrative model (Fig. 1), such as providing information on consequences of sustainable healthy eating behaviour; providing information about others’ approval or disapproval of proposed behaviour change; or prompt intention formation through encouraging a person to decide to act or set a general goal, e.g. one day per week without meat. Even though the ultimate goal of our intervention is to stimulate sustainable healthy eating behaviour, communication should create, change or reinforce specific beliefs [50]. Baker and co-authors [86] have shown that a focus on strengthening attitudinal beliefs (via changing beliefs about consequences) and boosting self-efficacy of young adults can result in higher intentions and actual behaviour towards eating healthier.

The intervention will take 1 month. The six behaviours introduced in the campaign “Livewell 2020”: eat more plants, waste less food, eat less meat, eat less processed food, eat variety of food, and eat more certified food will be explored during the interviews, adapted to the Polish context and communicated to consumers. The topics will be also refined based on findings from Studies 1 and 2. At the end of the intervention data for all of the users will be provided with the content of the messages that were sent and information whether the participants clicked through to read the page and how much time they spent on the page. After the intervention participants will complete a follow-up survey consisting of similar variables as the baseline survey. The interaction with participants through the mobile application is expected to bring people to a desirable more rational and conscious behaviour as suggested on Fig. 1. All participants will be volunteering adults and will provide informed consent. The study will be submitted for clearance to the local ethical committee.

Subsequently all data, also the data from the questionnaires, will be analysed using statistical software programs: SPSS 22.0 and LISREL 9.1. All statistical tests will be two-sided at the 5 % significance level. All treatment evaluations will be performed on the principle of intention-to-treat (ITT), using the observed data collected from all randomised participants. Appropriate imputation methods will be applied to the missing data on the primary outcome. A cluster analysis will be used to identify consumer segments based on their knowledge, attitudes and dietary habits. Structural equation modelling (SEM) will be applied in order to evaluate the effectiveness of the intervention, and particularly to explore the impact of different mediators and moderators (following the theoretical framework) on consumers’ behavioural intention and behaviour towards sustainable and healthy eating. SEM has not been extensively used in the experimental studies. The application of SEM in experimental studies represents a significant but relatively untapped potential area of application [87].

## Discussion

The project approach is novel and original both in terms of the nature of the research questions being addressed and the methodologies envisaged to be employed. To date sustainability and healthy eating have not been readily combined in consumer behaviour research. The importance of and need for developing interventions aiming at increasing consumers’ sustainable healthy eating behaviour through influencing their attitudes, self-efficacy and knowledge about the topic has been highlighted [24]. Secondly, the use of longitudinal qualitative research with three separate time periods is a relatively recent methodological approach that has yet to be fully articulated as a coherent methodology [72]. It embodies a range of in-depth interviews which involve returning to interviewees to measure and explore changes which occur over time and the processes associated with these changes [73]. This novel approach is very relevant for this research proposal as sustainable healthy eating behaviour is not explored yet in Poland.

Thirdly, the use of randomized controlled trial (RCT) in social and agricultural economic science; and particularly in consumer research is innovative. Most of the studies use cross-sectional data, where the causal effects between the mediators and endogenic variables cannot be proven. In the experimental setting, such as a RCT we will be able to measure the actual links between the variables. Fourthly, the application of structural equation modelling as a technique to evaluate the effectiveness of the intervention as well as the role of different mediators and moderators is new. Only few studies have successfully applied this method so far e.g. [88]. Finally, the use of new technologies, such as mobile learning for providing information to consumers is innovative as well.

The implementation of the principles of sustainable development focuses particularly on the sphere of food consumption. Incorrect eating habits may be harmful for human health and are proved to contribute to the development of (chronic) diet-related diseases among young consumers. Consumers’ behaviour related to food slightly supports the principles of sustainable development. Therefore, it seems reasonable to identify the determinants of consumer attitudes and behaviour in relation to sustainable healthy eating. Such formulated objectives of this project proposal are relevant in terms of social, health and environmental aspects. Sustainability and healthy eating issues are connected in an innovative way with the social objectives and principles of sustainable development.

## Abbreviations

BCT, behaviour change techniques; CAPI, computer assisted personal interviewing; RCT, randomised controlled trial; SEM, structural equation modelling; SHE, sustainable healthy eating
